# Current Status of* Aedes aegypti* Insecticide Resistance Development from Banjarmasin, Kalimantan, Indonesia

**DOI:** 10.1155/2018/1735358

**Published:** 2018-12-20

**Authors:** P. H. Hamid, V. I. Ninditya, J. Prastowo, A. Haryanto, A. Taubert, C. Hermosilla

**Affiliations:** ^1^Veterinary Medicine, Gadjah Mada University, Jl. Fauna No. 2, Karangmalang, Yogyakarta, Indonesia; ^2^Institute of Parasitology, Biomedical Research Centre Seltersberg, Justus Liebig University Giessen, Schubertstr. 81, Giessen, Germany

## Abstract

*Aedes aegypti* represents the principal vector of many arthropod-borne diseases in tropical areas worldwide. Since mosquito control strategies are mainly based on use of insecticides, resistance development can be expected to occur in frequently exposed* Ae. aegypti* populations. Surveillance on resistance development as well as testing of insecticide susceptibility is therefore mandatory and needs further attention by national/international public health authorities. In accordance, we here conducted a study on* Ae. aegypti* resistance development towards several often used insecticides, i.e., malathion, deltamethrin, permethrin, *λ*-cyhalothrin, bendiocarb, and cyfluthrin, in the periurban area of Banjarmasin city, Kalimantan, Indonesia. Our results clearly showed resistance development of* Ae. aegypti* populations against tested insecticides. Mortalities of* Ae. aegypti* were less than 90% with the highest resistance observed against 0.75% permethrin. Collected mosquitoes from Banjarmasin also presented high level of resistance development to 0.1% bendiocarb. Molecular analysis of voltage-gated sodium channel (*Vgsc*) gene showed significant association of V1016G gene point mutation in resistance* Ae. aegypti* phenotypes against 0.75% permethrin. However, F1534C gene point mutation did not correlate to* Ae. aegypti* insecticide resistance to 0.75% permethrin. Irrespective of periurban areas in Kalimantan considered as less densed island of Indonesia,* Ae. aegypti*-derived resistance to different routinely applied insecticides occurred. Our findings evidence that* Ae. aegypti* insecticide resistance is most likely spreading into less populated areas and thus needs further surveillance in order to delay* Ae. aegypti* resistance development.

## 1. Background

*Ae. aegypti* is the key factor in spreading and transmission of various infectious diseases such as Dengue Fever (DF), Chikungunya, Yellow Fever (YF), and most recently also Zika [1, 2]. Among these viral diseases, DF was classified as most important mosquito-borne disease in the world by WHO due to its wide global burden and associated consequences of economic losses in public health systems [3]. Indonesia is included as “red area” on current WHO risk map for DF together with almost all nations across equator line with tropical/subtropical climates [4]. Due to its large population, Indonesia is the most affected country in South East Asia.

Indonesia is an island chain located in equator which is bridging two continents, namely, Asia and Australia. Our investigated island in this study, Kalimantan or also known as Borneo, has more rural areas than urban/ periurban areas surrounded by wet-rain forest, palm oil plantations, and coal mining companies. Escalating progress of man-made environment influence has resulted in increasing human movements rapidly through modern transportation networks from different islands to less unpopulated islands within Indonesia. History on how* Ae. aegypti* mosquitoes were introduced and inhabited the island of Kalimantan is still unclear. A feasible scenario of spread by sailors and their ships in colonialism era might have played relevant role in successful colonization of mosquitoes, similar to what happened in the past in Philippines and Hawaii [5]. Since insect control mainly relies on chemical compound usage all over Indonesian archipelago, we evaluated here resistance status of* Ae. aegypti* populations to frequently insecticides which may reflect changes of ecological bases/habitats in Kalimantan. Current problems faced by Kalimantan are mainly massive deforestation due to expansion of housing areas, extension of new palm oil plantations, coal mining- and logging-activities [6]. Ecological changes in vegetation composition of tropical rain forests, creation of new plantation watering systems, and dam systems for logging economic activities are nowadays considered to have serious impact on epidemiology and spreading of various human vector-borne diseases. Clear example is evidenced by recent ecosystem destruction in Africa where use of dams and culture of rice in paddy-fields produced large expanses of water which are suitable breeding spots for mosquitoes and water snails, both relevant vectors of human diseases such as malaria and schistosomiasis in sub-Saharan Africa [7]. Deforestation is also evidently enhancing landscape becoming warmer and therefore much more hospitable for mosquito colonization [8]. Studies on malaria mosquito vectors evidently showed that deforestation and resulting impact on microclimatic environments greatly influenced life cycles of mosquitoes by reducing vector generation times, increasing reproductive rates, enhancing larval–pupa survival rates, and finally enhancing vector populations [7, 8].

Due to its flexible feeding, as well as breeding habitats,* Ae. aegypti* has rapidly evolved in last decades as extremely adapted species to human environments.* Ae. aegypti*'s synanthropic behavior is relatively closer to humans when compared to other* Aedes* species, e.g.,* Ae. albopictus,* and thus leading to significantly higher spread of* Ae. aegypti*-transmitted diseases [9]. Reducing* Ae. aegypti* populations and minimizing interactions with humans are the most reasonable way to control transmitted diseases because complete eradication of suitable vectors and/or viruses is rather unrealistic control approaches [10]. Furthermore, appropriate vaccines and therapies for most human diseases caused by arboviruses are still under development.

Insecticides utilization is considered as the most efficient tools in vector control programs. Insecticide applications can thereby vary from aerosol-space spraying, coils, lotions, clothes, or curtains embedded with certain active insecticide compounds and mass fogging to usage of larvicides in breeding waters. Consequences of national policy breakdown by usage of massive insecticide-based controls with the same active compounds might result in insecticide resistance development. As such,* Ae. aegypti* resistance development to commonly used insecticides has been reported from different countries worldwide such as Colombia [11], Brazil [12], Grand Cayman [13], Thailand [14], India [15], Malaysia [16], Mexico [17], and China [18]. Consistently to these findings, we previously reported on urban* Ae. aegy*pti resistance development in the cities Denpasar [19] and Jakarta [20] and evidencing increased resistance to commonly used insecticides in Indonesia.

Mechanism of arthropod insecticide resistance development is linked to several pathways which are basically involving metabolic mechanisms and target sites [21]. Main target site for pyrethroid/chlorogenic resistance is represented by mutations in voltage-gated sodium channel (*Vgsc*) genes of* Ae. aegypti* [21]. Detailed* Vgsc* profiling of* Aedes* has identified at least seven point mutations of insecticide knockdown (*kdr*) which can clearly lead to reduced sensitivity of arthropod sodium voltage channels to exposed insecticides [22]. Identification of* Ae. aegypti* specific* Vgsc* genes showing S989P-, I1011M/V-, V1016G/I-, F1269C-, and F1534C-point mutations is related to this type of resistance [13, 23–26]. At least two of these mutations, i.e., V1016G/I and F1534C, were responsible for pyrethroid resistance as examples in Asia [14, 23, 27] and Latin America [24, 28]. Synergistic resistance may also occur by additional point mutations in different locations of* Vgsc* genes of* Aedes* thereby increasing thousands of times resistance capacities mainly towards permethrin-derived insecticides [29].

Kalimantan is well-known endemic area for not only relevant arthropod-borne viral infections, i.e., DF and YF, but also for important arthropod-borne protozoa such as* Plasmodium* mainly transmitted by endemic Anopheline mosquito species. Chemical compounds of pesticides and insecticides are widely utilized in Kalimantan for several purposes. Such compounds are not only used for controlling malaria and DF vectors, but also for spraying hectares of plantations which is targeting agricultural pest insects. Incidence of resistance development may even occur simultaneously when control programs raised against insects transmitting diseases to humans coincide with control programs against plant pests. Consequently, resistance surveillance has to be performed periodically even in less populated (periurban) or agricultural areas to design strategies for avoiding or minimizing double insecticides exposure of local insect vectors. Although the city centre of Banjarmasin is also containing a relatively dense human population, several urban parts also show less density. Our sampling points were performed in periurban areas of Banjarmasin corresponding to less populated boroughs and further directly bordering forested areas. These sampling sites were in contrast with our previous sampling sites in Denpasar and Jakarta [20, 21], which all were allocated close to the city centre [19, 20]. In this study, we explored resistance development of* Ae. aegypti* in Banjarmasin, Kalimantan, to several types of commonly used insecticides. Our results provide first important information of current resistance status of* Ae. aegypti *populations in this investigated area.

## 2. Methods

### 2.1. Ethics Approval and Consent to Participate

No ethical clearance was requested as these experiments included exclusively* Aedes* mosquitoes. Moreover, no mosquito blood feeding was here used and only F0 specimens were used for all experiment settings. The allocation of ovitraps on residential housing areas represented any potential risk of infection neither for humans nor domestic animals and therefore no ethical consent was here issued.

### 2.2. Mosquito Samples

All mosquitoes in this experiment were collected from periurban areas of the city Banjarmasin (3°19′52.0′′S 114°36′29.3′′E) in South Kalimantan, Indonesia. These collection sites were designed to cover periurban areas of the northern (3°16′23.9′′S 114°35′16.8′′E), southern (3°21′46.0′′S 114°34′55.4′′E), and eastern (3°18′09.9′′S 114°36′42.6′′E) parts of the city. All mosquito samples were collected in July 2017. Ovitraps were placed after obtaining verbal permission from locals as performed in our experiments previously [19, 20]. Briefly, ovitraps were constructed from glass with a black staining outside. A filter paper was placed in the mouth of ovitrap glass and thereafter filled up to 3/4 with tap water. Filled tap water and filter papers were regularly replaced every week during collection dates. Egg-containing filter papers were carefully collected and dried at room temperature (RT), stored, and thereafter transported in plastic containers to the Gadjah Mada University in Yogyakarta, Indonesia. Mosquito eggs were allowed to hatch in the insectary of the Gadjah Mada University. Freshly hatched* Ae aegypti*-larvae were maintained in plastic containers with tap water daily fed with chicken liver (wet and dried) until reaching adult stage. All adult mosquitoes reared from these collected eggs were thereafter fed with a 10% sugar solution absorbed into cotton balls within specially designed entomological cages of the insectary. Emerged mosquitoes (F0) up to three days old were exclusively used for all insecticide-related experiments.

### 2.3. Applied Insecticide Susceptibility Tests (ITS)

Insecticide susceptibility tests (IST) were conducted according to the international approved WHO protocols for Anopheline mosquitoes diagnostic doses [30]. Briefly, the kits and insecticide-impregnated filter papers were prepared and supplied by the Vector Control Research Unit, University Sains Malaysia as officially WHO collaborating centre within Southeast Asia. Impregnated filter papers tested were containing 5% malathion, 0.05% deltamethrin, 0.75% permethrin, 0.05%  *λ*-cyhalothrin, 0.1% bendiocarb, and 0.15% cyfluthrin. In each IST assay, a minimum of 120-150 alive adult mosquitoes from three areas of Banjarmasin were divided into 6 tubes, each containing at least 20-25 mosquitoes. Four tubes (4 replicates) served as replicates for 1 insecticide exposure and two tubes were additionally used as controls. The mosquito mortality was calculated by percentage according to the following formula: total number of dead mosquitoes/total sample size X 100 as reported elsewhere [30]. Abbott's formula was not used in this study since control mortalities were always less than 5%. The tests were again performed in triplicate. A total number of mosquitoes here tested were 402 specimens for 5% malathion, 404 for 0.05% deltamethrin, 420 for 0.75% permethrin, 402 for 0.05%  *λ*-cyhalothrin, 420 for 0.1% bendiocarb, and 402 for 0.15% cyfluthrin. Some mosquito survivors as well as dead mosquitoes from these IST bioassays were kept at −20°C for further molecular resistance development analysis. Resistance status of mosquito populations was defined according to the WHO recommendation corresponding to a threshold of < 90% mortality [30].

### 2.4. DNA Isolation

Isolation of DNA was performed using PureLink® Genomic DNA Isolation Kit (Invitrogen) according to manufacturer instruction. Additionally, we added combination with occasional vortexing using glass beads to ease lysis of mosquitoes in the early step of DNA isolation. Dead and surviving mosquitoes obtained from IST bioassays were extracted individually.

### 2.5. V1016G and F1534C Genotyping

Genotyping of the mutants V1016G and F1534C was performed according to previous report in literature [14, 31] for allele-specific PCR assays. For detection of V1016G, the PCR consisted of 1 *μ*l of 10 pmol forward primer 5′ACCGACAAATTGTTTCCC3′, 0.5 *μ*l of 10 pmol of each reverse primer 5′GCGGGCAGGGCGGCGGGGGCGGGGCCAGCAAGGCTAAGAAAAGGTTAACTC3′ and 5′GCGGGCAGCAAGGCTAAGAAAAGGTTAATTA3′, 12.5 *μ*l of Dream Taq Green PCR Master Mix® (Thermo Fisher Scientific) in a 25 *μ*l total reaction volume. PCR reactions were performed as follows: 94°C at 2 min, 35 cycles of 30 s at 94°C, 30 s at 55°C, 30 s at 72°C, and a final elongation step for 2 min at 72°C. PCR amplification products were then loaded onto a 3% agarose gel. The F1534C detection PCR consisted of 1 *μ*l of 10 pmol forward primer 5′GCGGGCTCTACTTTGTGTTCTTCATCATATT3′, 0.5 *μ*l of 10 pmol of the forward primer 5′GCGGGCAGGGCGGCGGGGGCGGGGCCTCTACTTTGTGTTCTTCATCATGTG3′ and 1 *μ*l of reverse primer 5′TCTGCTCGTTGAAGTTGTCGAT3′, 12.5 *μ*l of Dream Taq Green PCR Master Mix® (Thermo Fisher Scientific) in a 25 *μ*l total reaction volume. Reactions were performed as follows: 94°C at 2 min, 35 cycles of 30 s at 94°C, 30 s at 60°C, 30 s at 72°C, and a final elongation step for 2 min at 72°C. PCR amplification products were then again loaded onto a 3% agarose gel.

### 2.6. Statistical Analysis

Statistical analysis and graphical presentation of obtained data were processed by using the commercial available software GraphPad Prism® 7.02. In order to determine mutations associated with resistance, phenotype and odds ratio estimation using recessive model with 95% confidence and Fisher's exact test were here performed, respectively. Chi square test was performed to evaluate Hardy-Weinberg Equilibrium (HWE). Differences were regarded as significant at the level of* P < *0.05.

## 3. Results

### 3.1. Adult Ae. aegypti Resistance Development to Tested Insecticides

**Figure 1:**
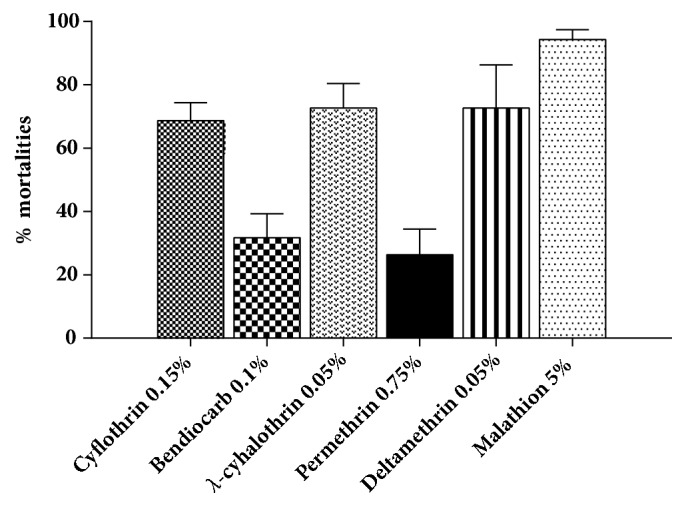
Resistance profiles to different insecticides tested in* Ae. aegypti* from Banjarmasin, Indonesia, 5% malathion (n= 402), 0.05% deltamethrin (n= 404), 0.75% permethrin (n= 420), 0.05%  *λ*-cyhalothrin (n= 402), 0.1% bendiocarb (n= 420), and 0.15% cyfluthrin (n= 402). The bars are percentage of mortality after exposure to insecticides with error bars represents standard deviation.

All adult mosquitoes (*n* = 2450) reared in laboratory from different locations of Banjarmasin were tested against various insecticides impregnated papers according to WHO protocols [30]. Based on IST results, all* Ae. aegypti* populations showed various degree of resistance to insecticides with mortalities less than 90% except for malathion 5% (Figure 1). Resistance profiles to permethrin 0.75% and to bendiocarb 0.1% were less than 50% (Figure 1). The highest resistance development observed was against permethrin when compared to any other tested insecticides except bendiocarb 0.1% (0.05% deltamethrin* t* = 5.059,* P* < 0.05; 0.05%  *λ*-cyhalothrin,* t* = 7.159,* P* < 0.01; 5% malathion* t* = 13.63,* P *< 0.001; 0.1% bendiocarb* t* = 0.831,* P *≥ 0.5; 0.15% cyfluthrin* t* = 7.42,* P* < 0.01).

### 3.2. Mosquito Genotyping of Voltage-Gated Sodium Channel (Vgsc) Gene

**Figure 2:**
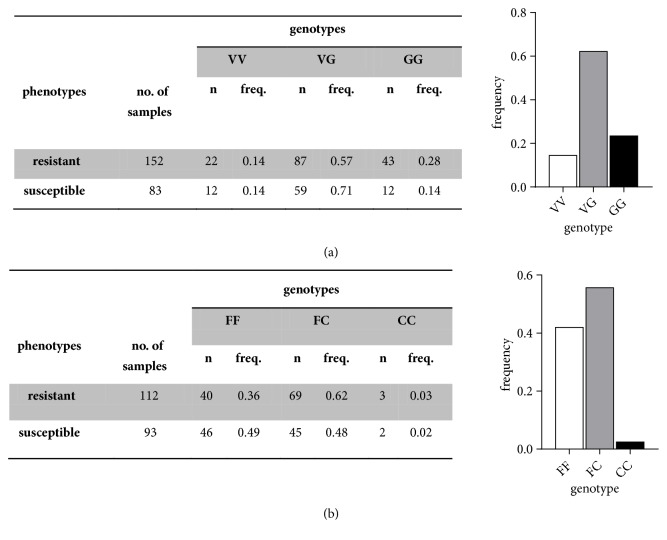
Distribution of V1016G and F1534C of* Ae. aegypti* in Banjarmasin. (a) VV, VG, GG and FF, FC, CC genotypes frequency.

PCR genotyping showed distribution of V1016G and F1534C in* Vgsc* gene domains II and III. In resistant mosquitoes, the wild-type VV homozygotes were 0.14, VG heterozygotes were 0.57, and GG homozygotes were 0.28. In susceptible group, VV frequencies were 0.14, VG were 0.71, and GG were 0.14. Distribution frequencies of total VV, VG, and GG genotypes in Banjarmasin were 0.145, 0.621, and 0.23, respectively. Total V1016 frequency was 0.455 and 1016G was 0.545, respectively (Figure 2(a)). HWE calculation of V1016 and 1016G showed* P* < 0.05. The descriptive distribution pattern of GG genotype in resistant and susceptible mosquito populations is provided in Table 1. Odd ratio (OR) estimation using recessive model of total GG genotype in V1016G mutations showed significant association (Fisher's exact test, P = 0.02, OR = 2.33, 95% CI = 1.16-4.59) with permethrin-resistance phenotypes (see Table 1).

F1534C distributions of F to C point mutations in* Vgsc* gene domain III were provided in Figure 2(b). In resistant phenotype, homozygotes frequencies of CC were 0.03 and in heterozygotes FC frequencies were 0.62, respectively. Susceptible mosquito samples showed CC frequencies of 0.02 and of 0.48 for FC frequencies (Figure 2(b)). Distribution frequencies of total FF, FC, and CC genotypes in Banjarmasin were 0.42, 0.56, and 0.02. Total F1534 frequency was 0.698 and 1534C was 0.302 (Figure 2(b)). HWE calculation of F1534 and 1534C showed* P* < 0.05. The CC genotype was rarely detected in* Ae. aegypti* populations originating from Banjarmasin. Odd ratio (OR) of F1534C mutation showed no significant association (Fisher's exact test,* P* = 1, OR = 1.25, 95% CI = 0.25-7.17) with phenotypes tested against permethrin (Table 1.). Heterozygotes were widely distributed in* Ae. aegypti* population compared to homozygotes of both wild-type and mutant alleles.

## 4. Discussion

In contrast with our previous reports from other Indonesian cities [19, 20],* Ae. aegypti* population in Banjarmasin was still susceptible to 5% malathion since mortality rate achieved more than 90%. Surprisingly, resistance to bendiocarb was as high as for permethrin. The resistance to permethrin is among the highest corroborating previous reports conducted in Indonesia [19, 20, 32, 33]. The genotype frequency of V1016G in Banjarmasin was significantly deviated from HWE. Mosquitoes carrying heterozygote genotype of VG were higher when compared to previous research conducted in Indonesia. The genotype frequency of F1534C in Banjarmasin was also significantly departed from HWE. In accordance with other reports, the CC genotype is found rarely in the mosquito population in Banjarmasin [19, 20, 32, 33]. Considering that knockdown resistance is generally supposed to be a recessive trait [34], we used recessive model to evaluate the association of resistant phenotype with each single point mutation. Molecular analysis of voltage-gated sodium channel (*Vgsc*) gene showed significant association of V1016G gene point mutation in resistance* A. aegypti* phenotype against 0.75% permethrin. However, F1534C gene point mutation did not correlate to* Ae. aegypti* insecticide resistance development to 0.75% permethrin in this experiment.

Our results contribute with additional pivotal data of global development of resistance to insecticides occurring widely worldwide even in islands or geographic areas which were considered less exposed to overuse of insecticides. However, we speculate on possible insecticide cross-use against insects affecting crops and animals. As such, national and international oil palm companies in Kalimantan have used massively agrochemicals in past decades to boost oil production during critical palm culture periods exposed to various insects such as* Oryctes *spp.,* Tirathaba *spp.,* Thosea* spp., and species of order Lepidoptera. This phenomenon has also been reported in other geographic areas [35] where heavy use of insecticides occurred without selective management and thereby unnecessarily exposing synanthropic insects such as* Ae aegypti* mosquitoes.

Despite possibilities of insecticide cross-use between crops and animal/human-related insects, mosquito incursion from different areas is now becoming in focus. Several investigations evidently showed that* Ae. aegypti* can be found in airport entry points and/or seaports [36]. Additionally, wind-blown dispersal of various mosquito species has been demonstrated in the past. Since massive movements of good trades are increasing worldwide, especially by the use of online systems, these might also contribute in mosquito introduction in previously nonendemic areas and should be considered as risk factors by public health authorities. Nowadays everything can be bought via Internet and afterwards delivered with ease by international parcel services from large modern cities to far, less populated and remote areas. This phenomenon needs further attention since changes in trade mechanism through revolution industry 4.0 can not be avoided. Additionally,* Ae. aegypti* eggs are known to be highly resistant as dried stages and as such to be found in containers of ships crossing long oceanic distances and still surviving for more than a year. This survival strategy of* Ae. aegypti* allows this species to be transported with ease using global maritime, terrestrial, and air-borne travel and trade networks. Mosquito larvae can hatch easily once eggs become rehydrated in new place and to successfully complete their life cycle. All above-mentioned anthropogenic factors seem significantly to contribute in spread of infectious agents worldwide, crossing political borders, and boundaries. This study in Banjarmasin (Kalimantan/Borneo Island) also showed that distances and geographic barriers, e.g., straits and/or seas can not impede resistance development, as previously demonstrated in our studies in the cities of Denpasar (Bali island) [19] and Jakarta (Java Island) [20]. Another plausible scenario of resistance spread was the recolonization of certain species which occurred for instance in a region declared as being ‘mosquito-free geographic areas/nations' in Brazil and probably originating from areas in which mosquito eradication was never completely achieved [37]. We stressed the point that control programs have to be nation/international teamworks and furthermore to be coordinated simultaneously between regions across boundaries since insecticide resistance phenotypes/genotypes in a region may be introduced from other islands.

Putting all together, insecticide resistance-related investigations are needed for better understanding molecular based resistance mechanism and to assess resistance status in exposed mosquito populations and helping to reduce overuse of certain insecticides in national control programs. Public health local agencies may also consider combination of biological and nonbiological strategies in controlling these vectors. Additionally, impact of climate and geographic factors (e.g., altitude, temperature, annual precipitation, relative humidity, and biogeographic region) on mosquito-host interactions and spread of these mosquitoes into nonendemic areas of Kalimantan is relevant topics to be considered in future investigations in one of the most biodiversed islands of the planet.

## 5. Conclusions

Regular surveillance on insecticide resistance development of* Ae. aegypti* mosquitoes is mandatory to be performed and to cover all main islands of Indonesia to set better goals and allow proper evaluation of on-going mosquito control strategies. It is recommended that insecticide resistance on other disease-transmitting mosquito genera, such as* Anopheles* and* Culex*, shall be performed in parallel within this investigated area.

## Figures and Tables

**Table 1:** Association of V1016G and F1534C with resistance to 0.75% permethrin.

**type of mutation**	**phenotypes**	**genotypes**		**OR** **(95**%** CI)**	***p* value of Fisher's test**
		**VV and VG**	**GG**		
V1016G	resistant	109	43		
	susceptible	71	12	2.33	0.02 (S)

		**FF and FC**	**CC**	**OR** **(95**%** CI)**	***p* value of fisher's test**

F1534C	resistant	109	3		
	susceptible	91	2	1.25	1 (NS)

OR= odds ratio.

CI= confidence interval.

S= significant.

NS= nonsignificant.

## Data Availability

The data used to support the findings of this study are included within the article.
